# Correction: Effective coupling of rapid freeze-quench to high-frequency electron paramagnetic resonance

**DOI:** 10.1371/journal.pone.0235201

**Published:** 2020-06-18

**Authors:** The PLOS ONE Staff

The ORCID iD is missing for the first author. Author E. Gabriele Panarelli’s ORCID iD is: 0000-0001-8790-5328 (https://orcid.org/0000-0001-8790-5328).

The fourth author's name is spelled incorrectly. The correct name is: Edgar J. J. Groenen. The publisher apologizes for the error.
