# Supplementary material for: Effective coupling of rapid freeze-quench to high-frequency electron paramagnetic resonance
Source: PLoS One. 2020 May 11;15(5):e0232555. doi: 10.1371/journal.pone.0232555 (PMC7213726; doi:10.1371/journal.pone.0232555)
Supplement: S1 File — The data used for this article is publicly available on Figshare at the following link: 10.6084/m9.figshare.8982347. (DOCX) [file pone.0232555.s001.docx]

**Effective coupling of Rapid Freeze-Quench to High-Frequency Electron Paramagnetic Resonance**

E. Gabriele Panarelli, Peter Gast and Edgar J.J. Groenen

Department of Physics, Huygens-Kamerlingh Onnes Laboratory, Leiden University, PO Box 9504, 2300 RA Leiden, The Netherlands

*Supplemental material*

1. *Detailed description of the packing of RFQ samples for X-band EPR*

The preparation of RFQ samples for 9.5 GHz EPR was successfully standardized by Nami et al. (1). We followed this procedure with minor modifications. The RFQ samples are straightforwardly packed in quartz tubes, readily used as sample holders for 9.5 GHz EPR. The essential steps of this procedure, conducted in a polystyrene box filled with dry ice pellets, are:

- The quartz tubes (10 cm long, 3 mm inner diameter) are open on both sides, and are customized by tapering them on one side. This allows the accommodation of a polypropylene disk used as a filter.
- The tapered end is connected through a latex tubing to a hand-held 60-mL Norm-Jet disposable syringe used to create underpressure (instead of a water aspirator, as described in the original procedure).
- While manually creating underpressure in the syringe, the other side of the quartz tube is dipped in cold isopentane contained in a vial in contact with dry ice. The isopentane is thus aspirated through the tube, which is pre-cooled by it.
- By maintaining the underpressure in the syringe, the pre-cooled quartz tube is quickly transferred into the vial containing the RFQ sample in cold isopentane. This vial has previously been lain on dry ice to ensure thermal contact. By pushing the quartz tube to the end of the sample vial (and making sure that the filter-containing tapered part is always in contact with dry ice so as to prevent the sample from warming), the RFQ sample is sucked up the tube and accumulates through it thanks to the filter. With the settings of the RFQ apparatus described above, a 3-mm quartz tube is typically filled with 4 to 5 cm of sample.
- When all the isopentane contained in the sample vial has been aspirated, the latex tubing is cut and the quartz tube is stored in liquid nitrogen. As opposed to the procedure described in (2), the sample in the tube is not packed more tightly with a steel rod because of the relatively big amount of sample present in the tube, and because a tighter packing would result in a more difficult handling for applications at 275 GHz.

1. *Detailed description of the packing of RFQ samples for 275 GHz EPR*

The minuscule size of the capillaries used as sample holders for 275 GHz EPR (150 *µ*m inner diameter) poses a twofold problem. Firstly, accidental warming of the samples is easy and fast, in view of the tiny volumes involved. For this reason, since the warming of the samples has to be avoided at all costs, they have to be handled at cryogenic temperatures. This leads to the second issue, which is the difficulty of handling such small capillaries in a cryogenic atmosphere, while wearing cryoprotective gloves that reduce the user’s hand sensibility.

The procedure follows in essence the basic steps described in (2). The packing is carried out in a polystyrene box half-filled with liquid nitrogen. Thanks to a flow of cold nitrogen gas blowing on the surface of the liquid nitrogen, the average temperature in the box within the first 10 cm from the liquid nitrogen surface is kept below -100 °C.

- A home-built stainless-steel plate (15 × 15 cm) is placed on top of an octagonal polystyrene box (14 × 14 × 6 cm). The plate has a central hole (6 cm diameter) that accommodates an agate mortar (whose surface sits at the same height as the plate), tightened with screws. This mortar is used to grind the RFQ samples under liquid nitrogen. The plate also features a grid-like array of perforated holes (2 mm diameter, separated by 1 cm), which allow a better thermal exchange with the liquid nitrogen beneath once the box is filled.
- The ensemble of plate, mortar, and octagonal box is placed in another, larger polystyrene box (29 × 25 × 24 cm), which is then filled with liquid nitrogen up to the level of the plate surface (3). In this way, also the octagonal box will fill with liquid nitrogen, and so will the mortar, which will always be immersed in it. It is important, prior to pouring the nitrogen, to wet the outside bottom of the octagonal box, so that a film of ice will form that keeps the octagonal box steady in its position during the procedure.
- A RFQ sample contained in a quartz tube is transferred from liquid nitrogen into dry ice pellets for a few minutes to ensure the softening of the content upon reaching a relatively higher temperature. In this way, after quickly transferring the quartz tube onto the plate contained in the liquid nitrogen box, it is possible to collect the RFQ sample in the form of pellets by tapping the surface with a pre-cooled glass capillary (2 mm outer diameter). This pellet of sample is then dropped in the mortar filled with liquid nitrogen with the help of another, smaller pre-cooled glass capillary (1.1 mm outer diameter) pushed through the first one. Two to four pellets are the necessary amount of sample to be ground and packed in a capillary for 275 GHz EPR.
- The pellets of sample are ground to a fine powder in the mortar filled with liquid nitrogen, by means of a pre-cooled agate pestle. Since the ground sample has the tendency to stick onto the surface of the mortar, it is important to scoop it with a pre-cooled metal spatula so as to stir it around and facilitate the packing procedure.
- A customized quartz capillary of 150 *µ*m inner diameter is used to collect the sample. Note that the customized capillary has a small tape flag that ensures the filter (and therefore the sample) to sit at a fixed position in the capillary, so that the sample will result in the middle of the insert’s resonant cavity. Also, it can be noticed that the capillary has an extra portion of it beyond the flag. This portion allows the capillary to be connected - through a plastic tubing - to a 60-mL Norm-Jet disposable syringe with a straight-cut Luer needle, used to manually create underpressure over the capillary and aspirate the powdered sample.
- Once the capillary is connected to the syringe, the underpressure made, and the capillary pre-cooled, the latter is dipped into the mortar, and by manually keeping the syringe piston tight, the sample is sucked up the capillary till the silica gel filter, where it accumulates. This is a critical step, because if the filter does not have specific value ranges of thickness and distance from the capillary bottom, the powdered sample will accumulate only at the tip of the capillary and get stuck there. This results in a gradual decrease of the underpressure, and further packing is made impossible.
- When at least 5 mm of the capillary have been filled with sample, the capillary is placed on a pre-cooled home-built metal block, whose function is that of helping keep the capillary at low temperature, protecting it from accidental warming. The extra portion of capillary connected to the syringe is cut, and the metal block is closed with its own lids, which are then fixed with screws. The metal block is then put in dry ice, and is ready for the loading. At least two to three capillaries are prepared per sample, because their accidental breaking or exposure to room temperature is easy during handling and loading.

*Bibliography*

1. Nami F, Gast P, Groenen EJJ. Rapid freeze-quench EPR spectroscopy: improved collection of frozen particles. Appl Magn Reson. 2016;47:643–53.

2. Nami F. PhD thesis, Leiden University; 2017.

3. Panarelli E.G. PhD thesis, Leiden University; 2018.
