# Supplementary material for: Effective coupling of rapid freeze-quench to high-frequency electron paramagnetic resonance
Source: PLoS One. 2020 May 11;15(5):e0232555. doi: 10.1371/journal.pone.0232555 (PMC7213726; doi:10.1371/journal.pone.0232555)
Supplement: Attachment — Submitted filename: Reviewing .pdf [file pone.0232555.s002.pdf]

Reviewing of :

Title : Effective coupling of Rapid Freeze-Quench to High-Frequency Electron Paramagnetic Resonance

Authors : E. Gabriele Panarelli, Harmen van der Meer, Peter Gast and Edgar J.J. Groenen

General comments : The authors present an original manuscript about coupling preparation of samples using Rapid Freeze-Quench to High-Frequency Electron Paramagnetic Resonance.

Preparing samples for high-frequency EPR is often challenging specially in the case of biologic systems when one wants to obtain reactive intermediates and/or follow kinetics (and it is also true for lower EPR frequencies). Here, samples have to be made in very small capillaries, with the difficulties related to its *possible* rapid warm and then degradation of the intermediate. Here, the authors use a classical reaction, *i.e.* the binding reaction of azide to myoglobin, to demonstrate the feasibility of the method. Globally, the article is well written although the technical part of filling the samples for both EPR frequencies could me better explained, and could be illustrated by a scheme. The article is very interesting, and will be of great help for researchers interested in using high-field EPR for their studies. I still have some comments/questions to address to the authors.

Specific comments :

1. line 80. How the authors determine the concentration of a frozen solution by absorption spectroscopy ?

2. Concerning the section "sample packing for 9.5 GHz EPR", it is not easy to follow if you don't have in mind the home-build apparatus. So I suggest that the authors prepare an illustration (scheme/figure) for the sample packing for both EPR frequencies.

3. In figure 1, the blue part, that I guess is the filled capillary, is not mentioned in the figure caption. I think also it would be useful and more understandable to have the different parts A/B/C in different colors.

4. line 179. The authors mention the Mn^{2+} signal obtained at high-field, but they do not explain why they need it, it is said latter in the text. I understand it is the internal standard. I think, since it is mentioned here (and before in line 77), it would be useful for the reader to have an explanation.

5. line 210. I do not think that the word "normalization" is correct in the sentence. It would be more correct to use the term "quantification of the conversion from HS -> LS signal), especially because at the end of the reaction, there is still a little bit of HS signal (the reaction is not complete).

6. starting at line 212. the mathematical demonstration is missing precisions concerning the different terms used : what is Y_t ? S_{HS} and S_{LS} ? In the reference 5, the demonstration is done in SI. I think it would be useful here to do the same for a better understanding of the article for the reader.

7. In figure 2, it would be useful for the reader to mention the sample names (Mb1 .... Mb10) in the figure together with the time. Could the authors mention the temperature of the experiment in the figure cation ?

8. line 218. The "intensities" word is not correct, it would be more appropriate to use "amplitude".

9. line 228. Could the contaminant be O_2 signal ?

10. When the authors talk about intensity, could they specify if it is "spin intensity" or "amplitude" ?
