# Supplementary material for: Effective coupling of rapid freeze-quench to high-frequency electron paramagnetic resonance
Source: PLoS One. 2020 May 11;15(5):e0232555. doi: 10.1371/journal.pone.0232555 (PMC7213726; doi:10.1371/journal.pone.0232555)
Supplement: Attachment — Submitted filename: RFQ Response PLOS rev.docx [file pone.0232555.s003.docx]

Response to the specific comments of the reviewer.

1. **line 80. How the authors determine the concentration of a frozen solution by absorption spectroscopy ?**

To determine the concentration of myoglobin, the absorption of the myoglobin solution (before, not after RFQ mixing) was measured at room temperature. To avoid this misunderstanding, we have changed the order of the sentences in the “Materials” paragraph.

2. **Concerning the section "sample packing for 9.5 GHz EPR", it is not easy to follow if you don't have in mind the home-build apparatus. So I suggest that the authors prepare an illustration (scheme/figure) for the sample packing for both EPR frequencies.**

The new aspect of the present study concerns the development at 275 GHz. The procedure at 9.5 GHz has been described in ref. 8, to which we refer at the beginning of this section, and an extensive description is given in the supplementary information. In the main manuscript we nevertheless summarized the procedure at 9.5 GHz, because the samples packed for 9.5 GHz form the basis for the packing at 275 GHz. We agree with the reviewer that this truncated description does not help. We have replaced it by a short paragraph that is easy to follow and keep the detailed description for the supplementary material.

3. **In figure 1, the blue part, that I guess is the filled capillary, is not mentioned in the figure caption. I think also it would be useful and more understandable to have the different parts A/B/C in different colors.**

We have described figure 1 more extensively in the legend. To add more colors did not improve the contrast and does not make it easier to recognize the different parts.

4. **line 179. The authors mention the Mn2+ signal obtained at high-field, but they do not explain why they need it, it is said latter in the text. I understand it is the internal standard. I think, since it is mentioned here (and before in line 77), it would be useful for the reader to have an explanation.**

We have corrected this by changing the order of the paragraphs of the “EPR measurements” section.

5. **line 210. I do not think that the word "normalization" is correct in the sentence. It would be more correct to use the term "quantification of the conversion from HS -> LS signal), especially because at the end of the reaction, there is still a little bit of HS signal (the reaction is not complete).**

We agree and have changed the wording accordingly.

6. **starting at line 212. the mathematical demonstration is missing precisions concerning the different terms used : what is Yt ? SHS and SLS ? In the reference 5, the demonstration is done in SI. I think it would be useful here to do the same for a better understanding of the article for the reader.**

We agree that the explanation in relation to equations (3) to (6) had to be improved. We have completely adapted the description.

7. **In figure 2, it would be useful for the reader to mention the sample names (Mb1 .... Mb10) in the figure together with the time. Could the authors mention the temperature of the experiment in the figure cation ?**

We have changed the legend to figure 2 accordingly.

8. **line 218. The "intensities" word is not correct, it would be more appropriate to use "amplitude".**

We agree and have changed to “amplitudes”.

9. **line 228. Could the contaminant be O2 signal ?**

We do not think so. A molecular oxygen signal is not to be expected at these field values.

10. **When the authors talk about intensity, could they specify if it is "spin intensity" or "amplitude" ?**

To avoid possible confusion, we use “amplitudes”.
